# Characterization and Predictive Value of Near Infrared 2-Deoxyglucose Optical Imaging in Severe Acute Pancreatitis

**DOI:** 10.1371/journal.pone.0149073

**Published:** 2016-02-22

**Authors:** Cristiane de Oliveira, Krutika Patel, Vivek Mishra, Ram N. Trivedi, Pawan Noel, Abhilasha Singh, Jordan R. Yaron, Vijay P. Singh

**Affiliations:** 1 Department of Medicine, Mayo Clinic, Scottsdale, Arizona, United States of America; 2 Department of Medicine, University of Pittsburgh, Pittsburgh, Pennsylvania, United States of America; University of Szeged, HUNGARY

## Abstract

**Background:**

Studying the uptake of 2-deoxy glucose (2-DG) analogs such as 2-Deoxy-2-[18F] fluoroglucose (FDG) is a common approach to identify and monitor malignancies and more recently chronic inflammation. While pancreatitis is a common cause for false positive results in human studies on pancreatic cancer using FDG, the relevance of these findings to acute pancreatitis (AP) is unknown. FDG has a short half-life. Thus, with an aim to accurately characterize the metabolic demand of the pancreas during AP in real-time, we studied the uptake of the non-radioactive, near infrared fluorescence labelled 2-deoxyglucose analog, IRDye^®^ 800CW 2-DG probe (NIR 2-DG; Li-Cor) during mild and severe biliary AP.

**Methods:**

Wistar rats (300 g; 8–12/group) were administered NIR 2-DG (10 nM; I.V.). Mild and severe biliary AP were respectively induced by biliopancreatic duct ligation (DL) alone or along with infusing glyceryl trilinoleate (GTL; 50 μL/100 g) within 10 minutes of giving NIR 2-DG. Controls (CON) only received NIR 2-DG. Imaging was done every 5–10 minutes over 3 hrs. Average Radiant Efficiency [p/s/cm²/sr]/[μW/cm²] was measured over the pancreas using the IVIS 200 *in-vivo* imaging system (PerkinElmer) using the Living Image^®^ software and verified in *ex vivo* pancreata. Blood amylase, lipase and pancreatic edema, necrosis were measured over the course of AP.

**Results:**

NIR 2-DG uptake over the first hour was not influenced by AP induction. However, while the signal declined in controls and rats with mild AP, there was significantly higher retention of NIR 2-DG in the pancreas after 1 hour in those with GTL pancreatitis. The increase was > 3 fold over controls in the GTL group and was verified to be in the pancreas *ex vivo*. *In vitro*, pancreatic acini exposed to GTL had a similar increase in NIR 2-DG uptake which was followed by progressively worse acinar necrosis. Greater retention of NIR 2-DG *in vivo* was associated with worse pancreatic necrosis, reduced ATP concentrations and mortality, which were not predicted by the blood parameters.

**Conclusion:**

*In-vivo* fluorescent imaging of a non-radioactive near infrared 2-DG optical probe can predict the AP severity early during the disease.

## Background

While the utility of 2-deoxy glucose (2-DG) analogs is established in diagnosis and prognostication of malignant diseases, the relevance of such studies in inflammatory diseases is emerging [1,2,3,4,5]. Acute inflammatory diseases such as acute pancreatitis (AP) have a sudden onset with a rapid, variable and unpredictable course ranging from resolution with minimal care over a few days, to a severe course (severe acute pancreatitis; SAP) progressing to extensive pancreatic necrosis, requiring intensive care, a prolonged hospitalization with high costs and sometimes resulting in death. However we currently lack tools which can reliably predict the course of AP takes early on in the disease.

There are reports of increased 2-Deoxy-2-[18F] fluoroglucose (FDG) uptake in human pancreatitis detected by positron emission tomography (PET) imaging [6,7,8,9,10,11]. These are typically done with intent to study pancreatic cancer, and pancreatitis is an incidental finding contributing to the “false positives” seen. However, the relevance of the uptake of 2-DG tracers in predicting the AP severity early in the course of the disease is unknown and may need further exploration. Such evidence, if present in preclinical studies, can potentially guide studies in human pancreatitis.

The current project was designed to explore the behavior of, and study whether a 2-DG probe can be used to predict the severity of AP early in its course. For this we chose the non-radioactive, near-infrared probe (IRDye^®^ 800CW 2-DG; NIR 2-DG; Li-Cor) and studied its uptake into the pancreas during mild AP and SAP. NIR 2-DG has been previously used as a 2-DG mimetic for both *in vivo* and *in vitro* studies [12,13]. Compared to FDG it offers the additional advantage of not being limited by a short half-life, and since it is non-radioactive, it is amenable to high throughput imaging requiring less specialized instrumentation. The emission spectra of NIR probes range between 650–900 nm which allows for their *in vivo* use without interference by either absorbance or non-specific emission from neighboring tissue. The severe and mild AP models used in this study were chosen based on their relevance to human disease [14]. The mild model involves bilio-pancreatic duct ligation, which is sufficient to fulfil the criteria of biliary acute pancreatitis, but does not result in severe necrosis as shown recently [15], this is similar to mild biliary pancreatitis as may occur in humans due to a mass in the head of the pancreas causing duct obstruction. The severe model simulates severe biliary pancreatitis complicated by fat necrosis, as may occur in humans [16,17,18,19,20,21]. In this, the triglyceride precursor of the second most abundant unsaturated fatty acid in pancreatic fat (linoleic acid), i.e. glyceryl trilinoleate (GTL) is infused into the pancreatic duct prior to the ligation. The amount of GTL is equivalent or lower than the percentage of adipocytes in the obese human pancreas [14,22,23,24,25,26]. Using the above tools we set out to study whether the course of NIR 2-DG uptake in the pancreas in controls, mild and severe AP can be used to predict AP outcomes.

## Materials and Methods

### Reagents

IRDye 800CW 2-DG (NIR 2-DG, Li-Cor Biosciences) was dissolved in 1 ml sterile saline at a final concentration of 100 nM. Glyceryl trilinoleate (GTL; TCI America) for in vitro studies was dissolved and sonicated in HEPES containing buffer; for *in vivo* studies was directly injected into the pancreatic duct of the rats. Ketamine Hydrochloride (Ketaset, Fort Dodge Animal health), Xylazine (Anased, Lloyd Laboratories) and Isoflurane USP (Piramal Healthcare).

### Animal work

All experiments were approved by the Institutional Animal Care and Use Committee of the University of Pittsburgh (Pittsburgh, PA), the Mayo Clinic (Scottsdale, AZ) and the Animal Care and Use Review Office (ACURO), a component of the USAMRMC Office of Research Protections. Mice: 6–8 week old male CD-1/ICR mice (Charles River Laboratories, Wilmington, MA) housed with a 12-hour light/dark cycle at temperatures ranging from 21–25°C, were fed standard laboratory chow and allowed to drink ad libitum. Rats: 250–350 g male Wistar rats (Charles Rivers Laboratories, Wilmington, MA) were housed with a 12 hour light-dark cycle, fed standard laboratory chow and were allowed to drink ad libitum. A Jugular venous catheter was placed in all rats under anesthesia (ketamine-Xylazine, 90 mg/kg, 8 mg/kg respectively, I.P.; or isoflurane, 3% + 0.8–1 L/min induction; 1.5% + 0.8–1 L/min maintenance). The rats were left to recover for at least 3 days prior experiments. Catheters were flushed with 4U/ mL heparin/saline solution every other day and the patency of catheter verified.

### Acinar Harvest and 2-DG *in vitro* assay

For NIR 2-DG *in vitro* studies, pancreatic acini were harvested from mice and used in oxygenated HEPES buffer pH 7.4 as described previously [27,28,29]. Viability was confirmed by trypan blue exclusion (>95%) at the start of each experiment. All studies were done in a shaking water bath (80 RPM) at 37°C at an ambient atmosphere. Cells were treated with either glyceryl trilinoleate (GTL, 300 μM) or saline as a control. Cell injury was quantified by measuring LDH leakage (Roche Diagnostics, Indianapolis, IN) into the medium as described previously [22,30,31]. This was done at 0, 1, 2, 4 hours by taking a 2% (40 μl out of 2 ml) aliquot of medium, centrifuging it (200 g, 5 minutes) and measuring LDH in the supernatant. At the end of the incubation the cells were lyzed by incubating them for one hour in Triton x-100 at final concentration of 1%. LDH activity in this lysate was taken as representing total (100%). The activity in the aliquots calculated as a percentage of the total amount was depicted as % LDH leakage for each time point. For measurement of NIR 2-DG uptake, IRDye 800CW 2-DG (NIR 2-DG) was added at a final concentration of 1 μM to the medium containing the acini. These were then stimulated with GTL or saline. The reaction was terminated at 1 hour by putting cells on ice followed by 3 washes in ice cold HEPES buffer to remove unbound dye. The pellet was suspended in HEPES buffer and platted in triplicate in 96-well black plate. The plate was scanned at 800 nm for the targeted NIR 2-DG fluorescence signal (arbitrary units; AU) using the Odyssey Imaging System (Li-Cor) and analyzed using the Image Studio Software. Each condition in an experiment had a minimum of 3 replicates. Signals were averaged and the relative fluorescence was determined by normalizing to protein levels (mg/ml) per well measured using the BCA Protein Assay Kit (Thermo Fisher Scientific). Ratios were calculated by normalizing to the saline treated control group. 4 or more experiments were performed for each parameter.

### *In vivo* 2-DG uptake and AP studies

*In vivo* 2-DG fluorescence imaging was performed with an IVIS Spectrum *in vivo* imaging system (PerkinElmer). All fluorescent images were acquired using identical illumination settings (EX/EM ICG filter, f/stop 2, field of views 22, binning 8), using 0.5 second of exposure time and analyzed using the Living Image^®^ software. Fluorescence emission was normalized to photons per second per centimeter square per steradian and expressed as average radiant efficiency [p/s/cm²/sr] / [μW/cm²]. All NIR fluorescent images were displayed in the same scale of fluorescent intensity. At the day of experiments, rats under ketamine-Xylazine anesthesia had the fur overlying the abdomen and back completely shaved, the catheter patency checked and intravenous bolus injected with 100 μl of 2-DG (10 nM) followed by 100 μl of saline to wash the catheter. Preliminary studies were done to study early intensity changes. In the abdomen, the only changes noted were rapid accumulation in the bladder after 1 minute in the supine position and after 10 minutes as a diffuse uptake in the posterior superior abdomen in the right lateral position. After 10 minutes of 2-DG signal stabilization, severe AP was induced by intra pancreatic duct injection of GTL (50 μl/100 g of body weight) followed by ligating of the bilio-pancreatic duct just proximal to its entry into the duodenum (GTL group). Mild AP was induced by bilio-pancreatic duct ligation alone (DL group). Control groups just received 2-DG. For imaging, sedated rats were placed inside the 37°C warmed IVIS chamber and images were taken every 5–10 min after NIR 2-DG administration before AP induction and over the 3 hours following this. When rats were euthanized and pancreas harvested for *ex vivo* imaging or gauging the severity of pancreatitis. Animals were treated in a humane fashion with close attention to pain control. This was via keeping them anesthetized with ketamine-Xylazine, (90 mg/kg, 8 mg/kg respectively, repeated as needed when animal could be aroused by toe pinch) over the period imaging studies were done and electively sacrificing them 1 or 3 hours post AP induction. The sedation prevented interference from movement artefact. For post-AP induction survival studies, animals were administered cefazolin (WG critical care, NJ, 100 mg/kg, intramuscular) for infection prevention, buprenorphine SR (ZooPharm, at 0.6 mg/Kg, S.C.) for pain management and normal saline (10 ml, subcutaneously) immediately after AP induction and allowed to recover. These animals were monitored continuously for signs of distress. Those noted to become moribund after AP induction (between 5 and 8 hours in GTL group, median 6 hours) were sacrificed with carbon dioxide anesthesia. Animals were sacrificed electively at 8 hours in the DL group. Serum, tissue samples were collected to study severity. Blood and pancreas tissue were harvested to analyze plasma lipase, amylase, alanine aminotransferase (ALT), bilirubin, and pancreas tissue edema and ATP levels and for pancreatic necrosis analysis as described previously [14,22,32]. Blood glucose levels were measured before and after 2-DG injection. There were 8–12 rats in each group for all quantified data.

### Biochemical assays

LDH (Roche Diagnostics, Indianapolis, IN), plasma Amylase, Lipase, ALT (Pointe Scientific Inc) were measured following the manufacturer’s instructions [14,22,32] on the FlexStation 3 Multi-Mode Microplate Reader (Molecular Devices) and total bilirubin (Pointe Scientific Inc) on the Eppendorf Biophotometer (Eppendorf). Blood glucose concentrations were measured by the glucose oxidase method using a FreeStyle glucose meter (Abbott Laboratories).

### Pancreas water content

Pancreata was weighed on an analytic balance (wet weight) and dehydrated by heating at 37°C overnight (dry weight). Water content was calculated according to the formula:

[(wet weight−dry weight)/wet weight] x 100 and expressed in total weight percentage.

### Pancreatic tissue ATP level determination

This was done as described previously [31,33]. A bioluminescent kit was used to measure ATP levels (Sigma-Aldrich, Saint Louis, MO) following manufacturer’s instructions. Briefly, pancreatic tissue was disrupted in tri-chloroacetic acid and EDTA containing buffer followed by appropriate dilution in Tris-EDTA buffer and application of a luminescent substrate. Luminescence was measured on a Promega Glomax 20/20 Luminometer and was normalized per milligram of protein determined using the BCA Protein Assay Kit (Thermo Fisher Scientific).

### Pancreatic necrosis

Whole pancreas paraffin section (5 micron) slides stained by hematoxylin & eosin were examined by a trained morphologist (CDO) blinded to the sample as described previously [14,22,31,32,33]. In brief, all pancreatic parenchymal area was imaged using the PathScan Enabler IV slide scanner (Meyer Instruments, Huston, TX) and images were evaluated for acinar necrosis as described previously[14,22,31,32,33]. Necrotic area and total acinar area were measured in pixels for each pancreas. Percentage necrosis was reported as a percentage of total area for each pancreas.

### Statistical analysis

This was done using the SigmaStat statistical package integrated into the graphic program SigmaPlot 11 (Systat Software Inc, San Jose, CA). All values, unless otherwise specified, are reported as mean ± SEM. Groups were compared by one way analysis of variance (ANOVA) versus controls. Pairs were compared using a two tailed Students t-test when the distribution was normal (Shaprio-Wilk test) or a Mann-Whitney test when failing the Shapiro-Wilk test. Differences were considered significant at a p value <0.05

## Results

### NIR 2-DG uptake in the pancreas is not affected early in AP induction

We started with intent to understand the dynamics of NIR 2-DG uptake into the pancreas and whether this could reliably discriminate between mild and severe AP. Since rats are commonly used in surgical models of biliary pancreatitis, we first chose to learn if NIR 2-DG could be reliably visualized in an area overlying the pancreas in 250–350 g rats during the initial 3 hours over which AP progressively gets worse. The blood glucose in these rats after overnight fasting and prior to infusion of NIR 2-DG was 177±9.1 mg/dl and did not change over the duration of the study in controls (182±25 mg/dl). 3 groups were studied: controls, severe AP; i.e. rats with GTL infusion which develop severe pancreatic necrosis [14], and mild AP; with duct ligation alone who have a much milder course [15]. We chose a lateral position since the stomach overlies the pancreas and to avoid the confounding effect of a variable amount of food present in the stomach of rats despite fasting. A right side down position was chosen since the pancreas tail extends to the left side and thus would not be covered by the liver.

**Fig 1:**
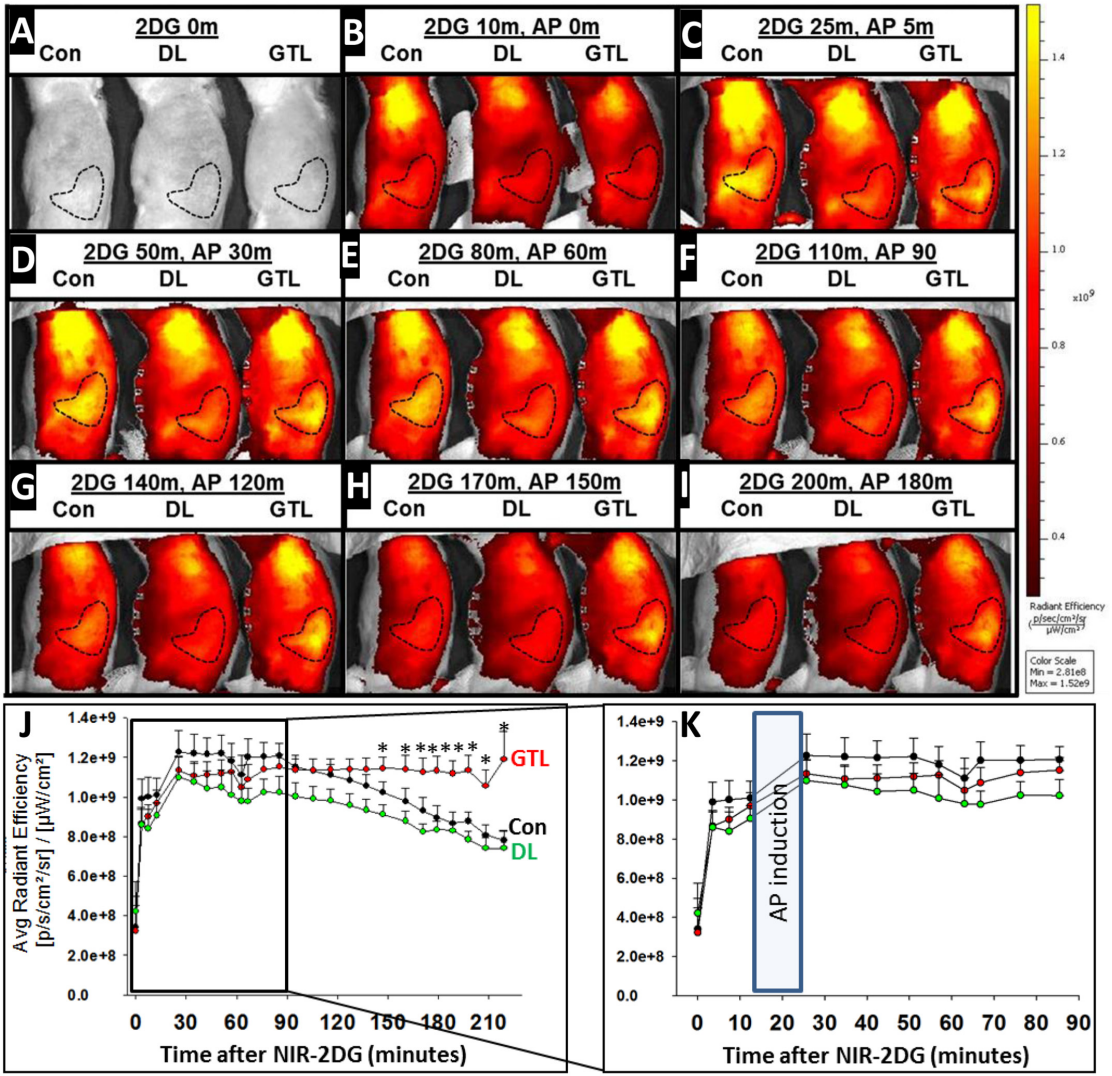
NIR 2-DG signal increase over the pancreas is unaffected by AP induction, but persists in severe AP unlike in mild AP or controls. **A-I:** Series of thoracic and abdominal images in the right lateral position acquired after different times in minutes (m) after administration of NIR 2-DF (2-DG) in controls, or after AP induction with duct ligation (DL) or GTL administration(GTL). The dashed polygon denotes the region of interest overlying the pancreas in which the fluorescence was measured. **J:** Average radiant efficiency as measured over time from when NIR 2-DG was administered in controls (Con, black circles), and in the duct ligation (DL, green circles) and GTL groups (red circles). There were 9–12 animals per group. * indicate the values that were significantly different from the corresponding controls at that time **K:** An enlarged view of the first 90 minutes showing the time when acute pancreatitis was induced.

Prior to NIR 2-DG administration, there was no fluorescence detectable in any group of sedated rats (Fig 1A). NIR 2-DG administration without AP resulted in an initial generalized increase in fluorescence which was similar in all groups (Fig 1B and 1K). This synchronously increased over the pancreas (dashed polygon fig 1A–1I) and plateaued by 30–40 minutes to values that were no different in the control (black dots Fig 1J and 1K), GTL and DL groups (red and green dots). This increase remained similar in all three groups for the hour following AP induction (Fig 1K) for a total of 90 minutes after NIR 2-DG administration. These results show that manipulation of the pancreas or induction of AP does not affect the initial uptake or equilibration of the NIR 2-DG.

### NIR 2-DG is retained in the pancreas during severe AP

**Fig 2:**
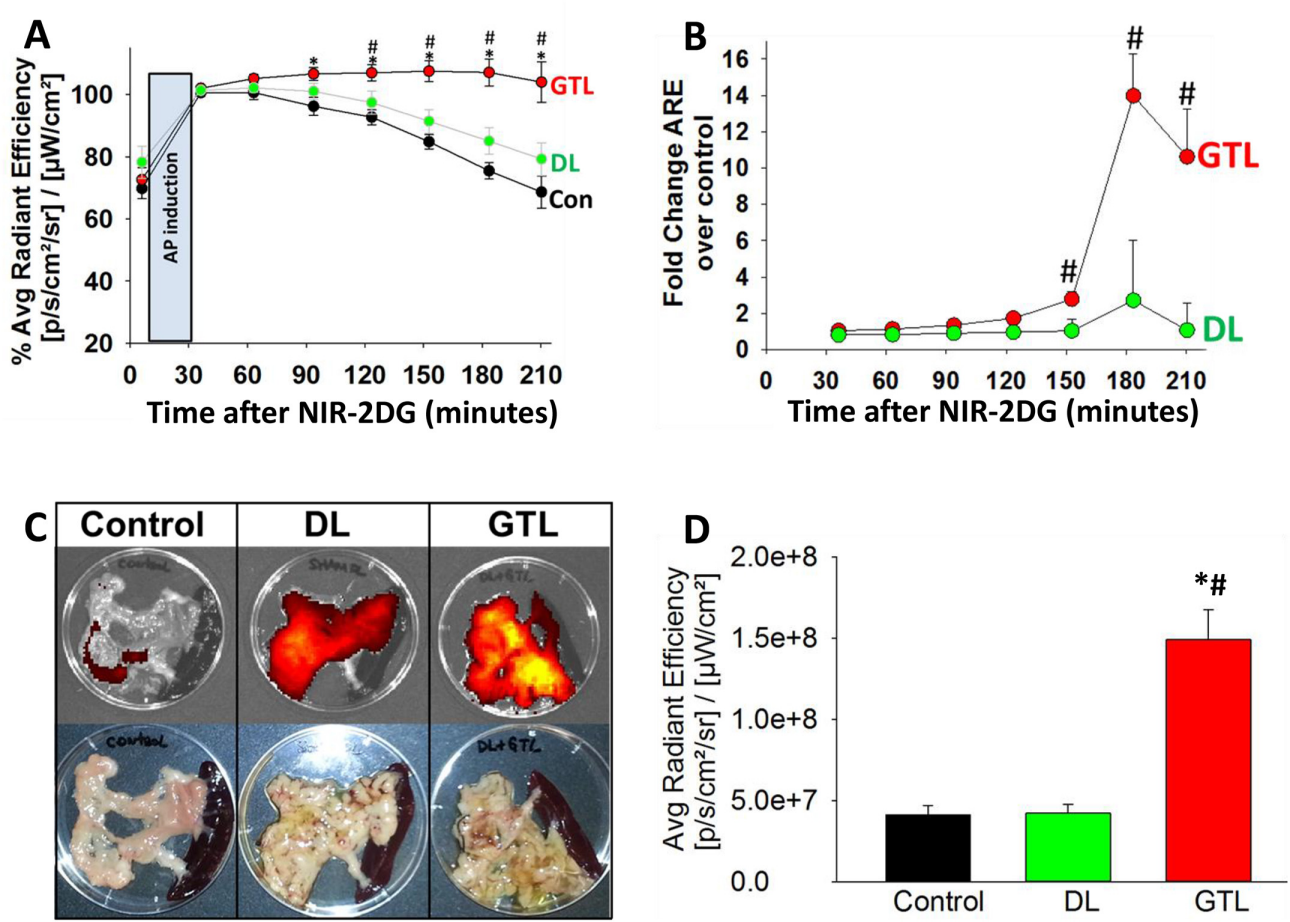
Severe AP is associated with significantly more retention of NIR 2-DG in the pancreas early during AP compared to controls. **A:** Time course showing means of the Average radiant efficiency for the values obtained ±15 minutes for each time point within each group (i.e. control; Con, duct ligation; DL, and GTL) after normalizing to the 30 minute peak (100%) for that group. * depicts a significant (p<0.05) increase in the GTL vs. control and # depicts significance vs. DL on ANOVA at each time point. **B:** Time course showing Average radiant efficiency (ARE) normalized to controls. The initial mean AREs for each group prior to AP induction, were subtracted from those at each time point. The difference in the DL or GTL pancreatitis groups was divided by the corresponding difference in the control group, and this ratio is shown as fold change over control. Please note, that with the decline in the ARE values in controls over time (A), there is a significant increase in this ratio in the GTL group vs. DL group (#). There were 9–12 animals per group. **C:** Fluorescence images (upper panel) and gross appearance (lower panel) of the pancreas *ex vivo* in the control, DL and GTL groups. **D:** Bar graphs showing the quantification of the ARE for each group. “*” depicts a significant (p<0.05) increase in the GTL vs. control and “#” depicts significance vs. DL on ANOVA.

We then analyzed the pattern of NIR 2-DG retention during the subsequent 2 hours. It was notable that during this time the control and DL groups progressively showed lesser retention of NIR 2-DG (Fig 1J). We then averaged the values for every 30 minute interval (i.e. time ± 15 minutes) and normalized it to the first 30 minute value when NIR 2-DG accumulation peaks or starts to plateau. As seen in Fig 2A the NIR 2-DG uptake in the GTL group remained elevated and equivalent to the peak 30 minute value for the subsequent 2 hours. This was significantly elevated over controls after 90 minutes (p<0.05, *, on ANOVA) and higher than the DL group at or after 120 minutes (P<0.03, #, Students t-test). To compare the fold increase in average radiance efficiency (ARE) in an AP group over the controls at any particular time point, the baseline ARE values i.e. those measured immediately after NIR-2DG administration (average 5 minutes) were subtracted from the ARE at that time point, and divided by the ARE change in controls. For e.g. the fold change in ARE at n minutes after NIR 2-DG in the GTL group was calculated as:

{ARE in GTL at n min.−ARE in GTL at 5 min.} ÷ {ARE in control at n min.−ARE in control at 5 min.}.

As can be seen in Fig 2B, this progressively increased, peaking at 3–14 folds between 2–3 hours of AP induction, while the increase in the DL group was insignificant. To verify that the increase was indeed in the pancreas, we removed the pancreas at 3 hours and measured the ARE overlying it. As seen in Fig 2C and quantified in Fig 2D, there was a large increase in the NIR-2DG accumulation in the pancreas of the GTL group compared to the DL group.

### Higher retention of NIR 2-DG may be helpful in predicting severe AP early on in the disease

**Fig 3:**
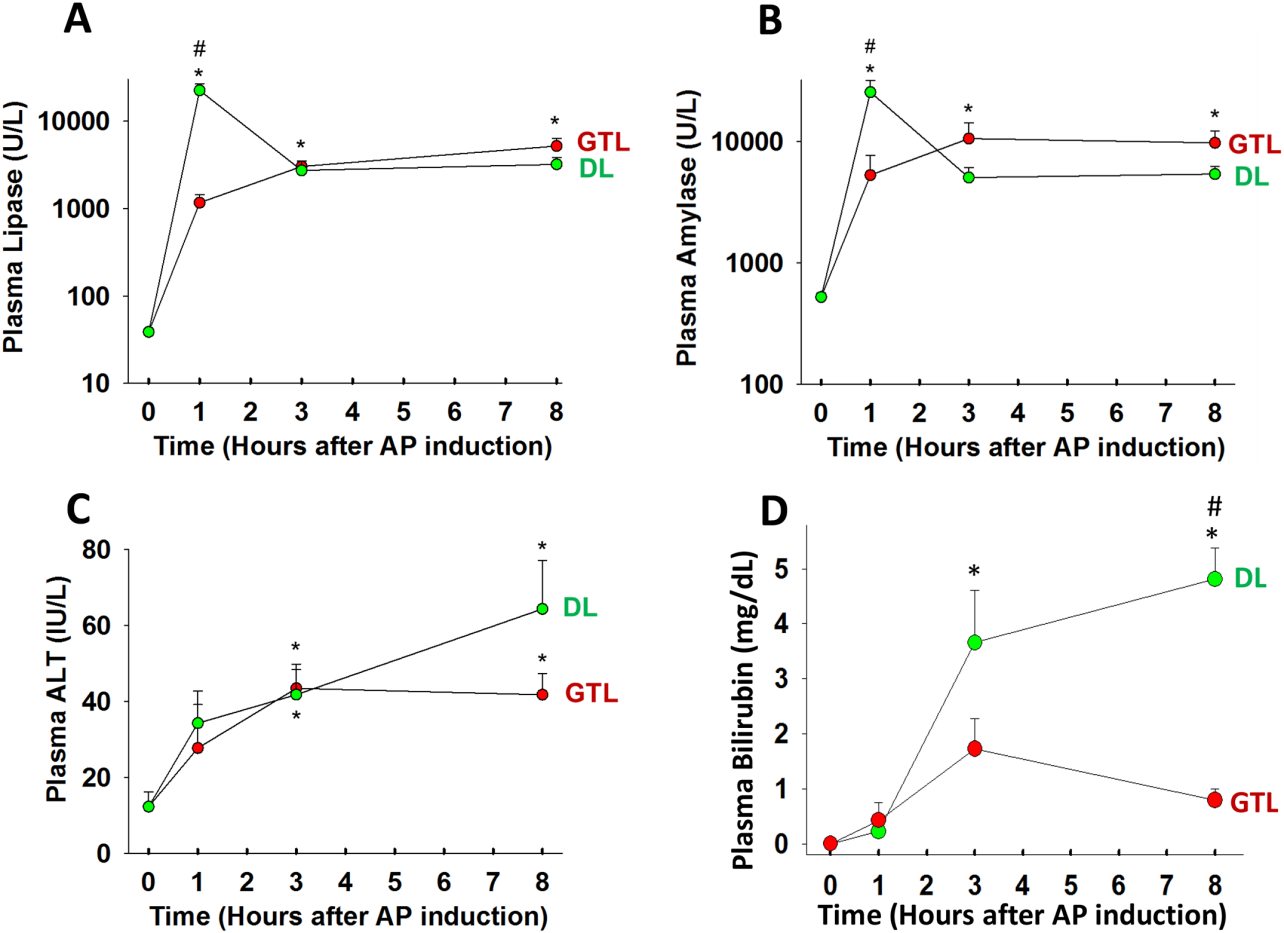
The increase of AP parameters in the blood is similar in both mild and severe disease. Time course of the activities of amylase (A), Lipase (B), alanine amino transferase (ALT; C) and amount of bilirubin (D) measured in heparinized plasma samples after 1, 3 hours of AP induction, at euthanasia (≤ 8 hours), and as baseline control value without the procedure (0 hours). Values at 1 hour, 3 hours and at the time of euthanasia for each group (DL- green, GTL-red) were compared to each other and the controls by ANOVA. “*” depicts a significant (p<0.05) increase vs. control and “#” depicts significance in GTL vs. DL. There were 8–12 animals per group.

We then compared the uptake of NIR 2-DG to parameters of AP commonly measured in blood. As can be seen in Fig 3A and 3B the parameters of AP induction i.e. plasma amylase and lipase were similar in the GTL and DL groups whether measured early or later in the disease course. Similarly the markers of a biliary etiology, i.e. plasma ALT and bilirubin were not higher in the in the GTL group. These findings correlate well with human data showing that criteria used to diagnose AP or its etiology have no bearing on its severity [34].

**Fig 4:**
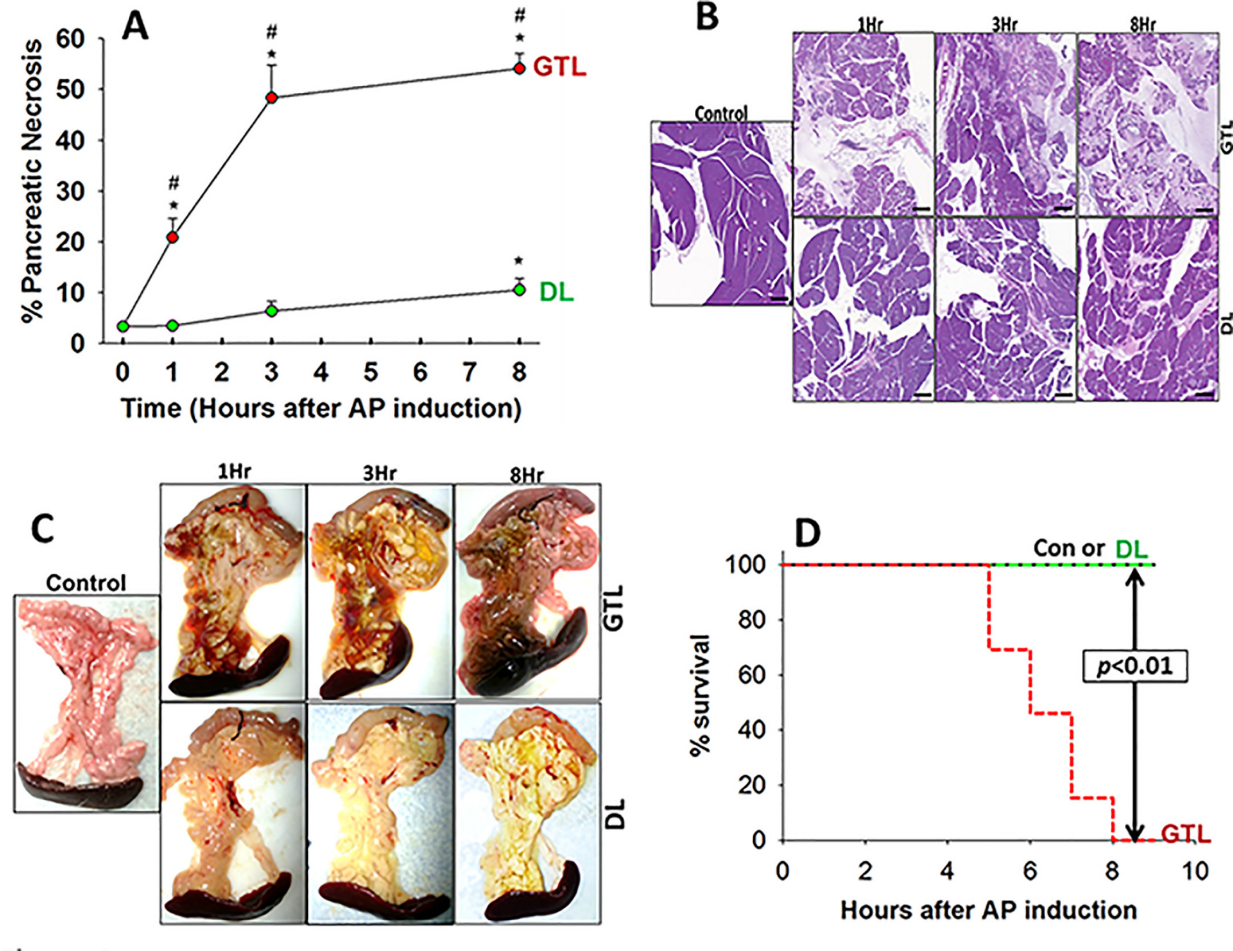
Severe AP is associated with a progressive increase in necrosis and mortality. A: time course of percentage of pancreatic parenchyma what was necrotic in the GTL (red) and DL (green) groups measured when electively sacrificed at 1, 3 hours and at euthanasia (≤ 8 hours). Values at 1 hour, 3 hours and at the time of euthanasia for each group were compared to each other and the controls by ANOVA. “*” depicts a significant (p<0.05) increase vs. control and “#” depicts significant differences in GTL vs. DL. Representative microscopic (5x, B) and gross (C) images are shown. D: Kaplan-Meyer curve showing % survival in the different study groups. There was 100% mortality in the GTL group by 8 hours, but none in the duct ligation group. There were 8–12 animals per group. Scale bars are 1mm in length.

**Fig 5:**
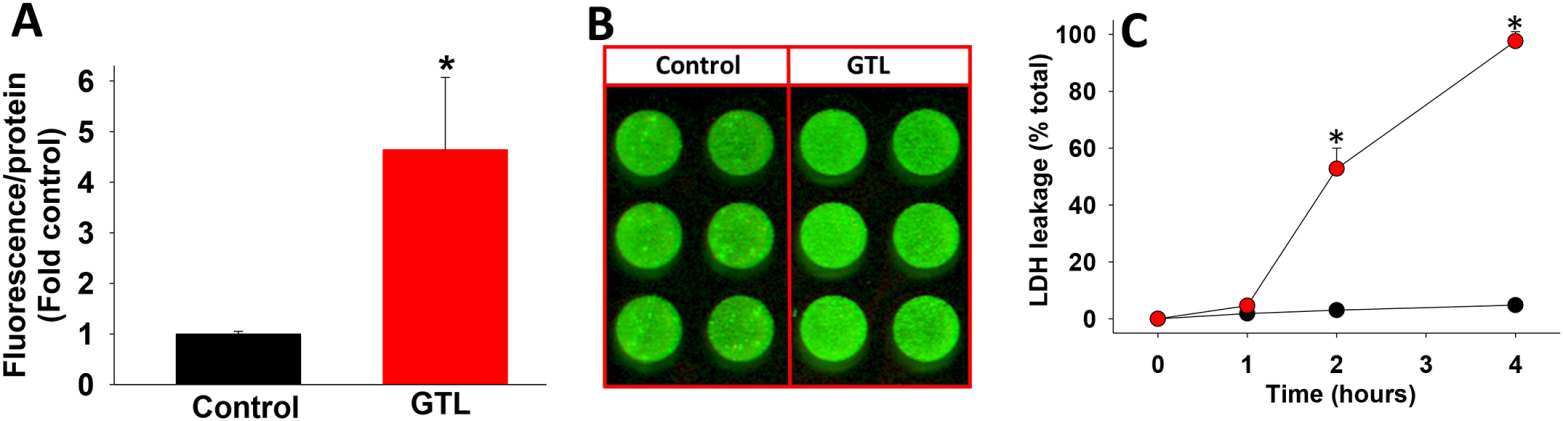
NIR 2-DG uptake *in vitro* occurs before the induction of GTL induced injury. Pancreatic acinar cells were exposed to 300 μM GTL and binding of NIR 2-DG (A) was measured at 1 hour, with results being depicted as fold control (arbitrary units fluorescence/mg protein). B shows an image of the bound fluorescence in control and GTL exposed acini as visualized on the Odyssey imaging system. C: shows the time course of LDH leakage induced by 300 μM GTL (red) vs. controls (black). The “*” depict *p*<0.01 vs. corresponding controls. Note, that the increased NIR 2-DG binding noted at 1 hour precedes LDH leakage. Data is representative of a minimum of 4 experiments.

**Fig 6:**
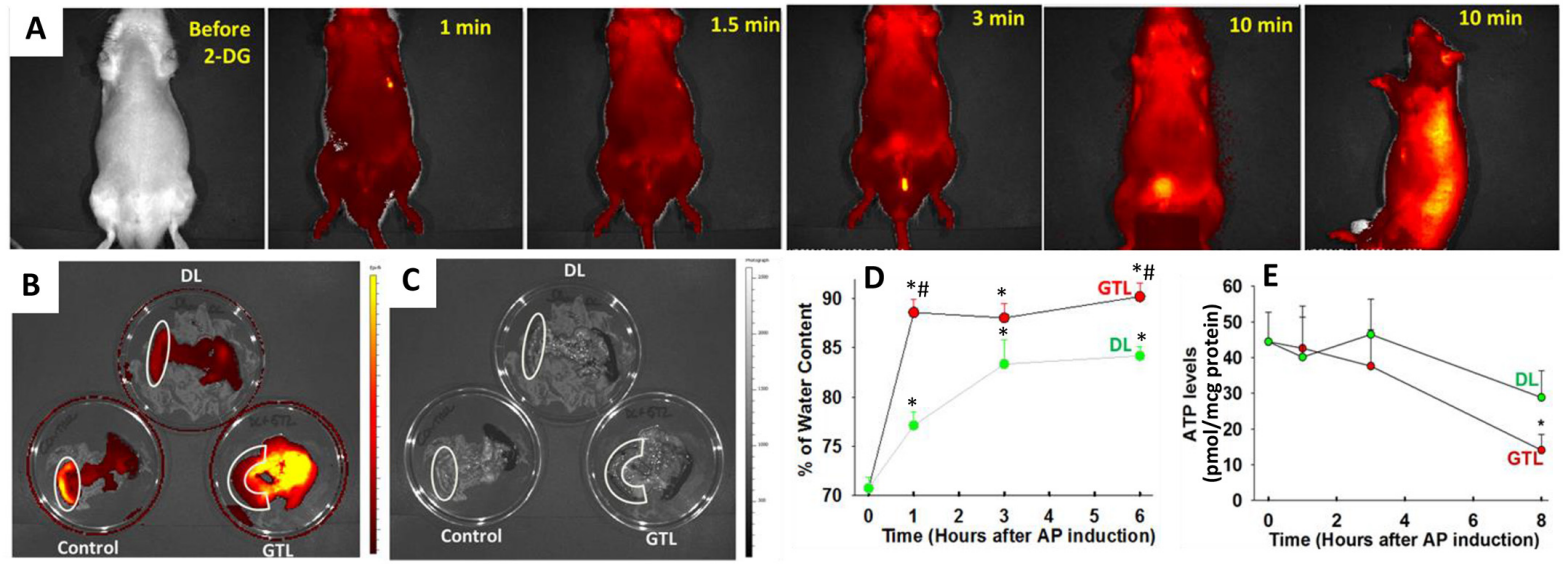
Parameters potentially affecting the NIR 2-DG signal measured in our system. **A:** Images of a rat administered NIR 2-DG collected on the IVIS imaging system in the supine position over the first 10 minutes (first five images) and in the right lateral position (last image). Note the increase over the bladder but the lack of signal over the pancreas in the supine position. This pattern is reversed in the right lateral position. Fluorescent (B) and black and white(C) images of the duodenum, pancreas and spleen removed *en block* after 2 hours of NIR 2-DG administration in controls or pancreatitis in the DL and GTL groups. Please note an increased uptake in the duodenum (white outlines) especially in controls. **D:** Data from groups of 8–12 animals showing the time course of the increase in pancreatic edema (measured as % water content or pancreatic ATP concentrations (**E**) in the DL and GTL groups. The “*” depict *p*<0.05 vs. corresponding controls “#” depicts significant differences in GTL vs. DL.

We then turned our attention to parameters of pancreatitis severity, focusing on local injury and mortality. Pancreatic necrosis in the GTL groups progressively increased from a median of 18% at 1 hour (Fig 4A–4C), when the necrosis in DL group was insignificant to a median of 54% (range 40–70%) at 8 hours with associated 100% mortality (Fig 4D). Duct ligation alone caused mild pancreatic necrosis at the time of sacrifice i.e. 8 hours (median 10%), at which time the pancreas had a yellowish hue, consistent with bile staining induced by the duct ligation. The worsening necrosis in the GTL group compared to the DL group correlates well with the fluorescence in the GTL group starting to increase between 60 and 90 minutes of AP induction (Fig 2A and 2B) and remaining increased over 3 hours. The *in vivo* findings of increased NIR 2-DG signal over the pancreas early in the course of AP were paralleled by increased NIR 2-DG binding to acinar cells within 1 hour of exposure to 300 μM GTL *in vitro* (Fig 5A and 5B). The fluorescence (arbitrary units/ well) /protein (mg/ml) was 54323 ± 18317 (AU/mg) in controls vs. 313265 ± 104180 AU/mg (*p*<0.01) in the GTL group. There was an insignificant increase in LDH leakage in the GTL group at this this point (4.6 ±1.7 vs. 1.8 ±0.4 in controls, p = 0.12), followed by a large increase in LDH leakage over 4 hours (Fig 5C). It is notable that the increase in NIR 2-DG uptake was associated with a trend of decreasing pancreatic ATP levels in the GTL group which became significant only in the group that was moribund prior to sacrifice (Fig 6E).

## Discussion

In this study we find that severe biliary AP is associated with an early and sustained retention of the near-infrared 2-DG probe (IRDye^®^ 800CW 2-DG; NIR 2-DG; Li-Cor) in the pancreas in rats. This retention is associated with worse pancreatic necrosis and mortality. The study is unique for the following reasons: **1)** It uses a rapidly evolving inflammatory disease model which is unlike the much longer duration cancer [12,13,35] or chronic inflammation [5,36] models commonly employing 2-DG probes. **2)** it shows the feasibility of detecting NIR signals in large (250–350 g) rats in contrast to mice which are commonly used for such imaging studies [12,13,37]. **3)** It shows that the increased retention of NIR 2-DG between 1 and 3 hours of AP induction has a stronger relation with severe outcomes than commonly used blood parameters used to determine AP severity in animal models.

Preliminary studies helped us decide the positioning of the rat and the timing of the imaging. While previous studies have shown a rapid first pass effect in mice with fluorescence overlying the kidneys to increase within 5 seconds of IRDye^®^ 800CW 2-DG administration [35], we did not note such an increase during the time fluorescence over the pancreas became intense (Fig 6A). We did note a rapid increase over the bladder to begin after the first minute and become intense by 10 minutes on imaging in the supine position. However we could not visualize the increase over the pancreas in this position, perhaps due to the stomach which overlies it. Placing the rat in a right lateral position allowed imaging the fluorescence over the pancreas (Fig 1A, dashed outline). The pancreatic signal was more cephalic and posterior than the bladder in the right lateral position. The right lateral position also resulted in the signal from the bladder to be largely masked by the pelvis and thigh, thus preventing it from interfering with the signal from the pancreas. In some cases we did note a distinct small signal from the bladder (seen in some images of Fig 1A–1I) which is anterior and inferior to the dashed outline corresponding to the signal from the pancreas. AP induction took 10–15 minutes and was executed within the 30 minutes that the signal peaked in controls (Figs 1K and 2A). This observation and the similarity in baseline glucose and other parameters between the 3 groups prior to AP induction prompted us to design the experiments to study 2 end points: **1)** whether AP induction affects the increase in signal from NIR 2-DG over the pancreas. **2)** Whether the pattern of NIR 2-DG signal over time can be used to predict the severity of AP.

As seen in Figs 1 and 2A, we note that AP induction does not interfere with the increase in ARE overlying the pancreas, which is similar in the controls and AP groups immediately after induction. After 30 minutes the trend changes with controls depicting a clear decline, whereas the GTL group shows a sustained increase over the course of the 3 hours these animals were imaged. The generalized increase over the posterior abdomen corresponding to where the kidney, pancreas, duodenum are located is at least partly contributed by the duodenum irrespective of AP as shown in the white outlines (Fig 6B and 6C). While bowel fluorescence could be easily excluded *ex-vivo* after removing the pancreas (Fig 2C and 2D) this could not be done *in vivo* since the imaging was planar. This uptake by the bowel likely contributed to the *in vivo* background signal in controls, making the signal from the GTL group comparatively less impressive. It remains to be seen whether such interference can be reduced by cross sectional imaging.

Lipolysis of GTL by pancreatic lipases results in the release of the unsaturated fatty acid linoleic acid. Human pancreatic necrosis collections have high concentrations of linoleic acid [14,31,38], which can result in necrotic cell death [39,40] via inhibition of mitochondrial complexes I and V [22]. This would also be consistent with the decrease in ATP levels we note in the damaged pancreas (Fig 6E). The amounts of GTL used in this study are less than the adipocyte mass in the obese human pancreas [14,22,23,24,25,26]. Further proof validating the GTL model as being representative of human severe biliary AP is discussed in the manuscript in which this was originally described[14]. Additionally, GTL was preferred over the bile salt infusion model since the concentrations of bile salts used (20–100 mM) are >10 times their critical micellar concentrations and thus have a detergent effect [41,42] on cell membranes. Moreover these high bile acid concentrations are in stark contrast to those in human pancreatic necrosis collections which are < 200 μM (unpublished data, not shown).

There are several factors that could be associated with increased NIR 2-DG retention during the progression of SAP. These include increased vascular permeability [43]measured as an increase in percentage water content of the pancreas during pancreatitis (Fig 6D), hemorrhage into the pancreas resulting in persistent NIR 2-DG accumulation, reduced clearance of NIR 2-DG in the GTL group, and hypoxia resulting in increased glucose requirement [5]. We have previously shown that lipotoxicity from unsaturated fatty acids such as linoleic acid causes a reduction in ATP levels associated with acinar necrosis [22,23], which would be consistent with the large increase in necrosis (Fig 4A–4C) and drop in ATP levels we note in GTL group (Fig 6E). These in combination with hypoxia could increase glycolysis dependence of the GTL treated pancreas and thus result in the increased NIR 2-DG uptake we note. The contribution of vascular permeability to the increased *in vivo* signal noted in the GTL group while possible is unlikely, since the fluorescence increase during pancreatitis *in vivo* (Fig 2) is similar to that in acini exposed to GTL *in vitro* (Fig 5A and 5B). Similarly, the increase in NIR 2-DG signal noted *ex-vivo* in the GTL group did not correlate with the amount or location of hemorrhages (Figs 2C, 6B and 6C), and while hemorrhages were sometimes noted within an hour of GTL injection (Fig 4C), these did not result in an increased of NIR 2-DG signal at 1 hour (Fig 1J and 1K). These observations along with the *in vitro* findings in acini mentioned above make the contribution of hemorrhage to the *in vivo* NIR 2-DG signal also unlikely. The possibility of variable clearance in affecting the NIR 2-DG signal is also unlikely since all animals were imaged at 5–10 minute intervals after its administration, and the signal increase was similar in all groups up to 90 minutes after administration. A selective reduction in clearance of NIR 2-DG the GTL group should have resulted in a generalized increase in NIR signal, but the increase noted *ex-vivo* was only over the pancreas and not the duodenum or spleen (Figs 2C, 6B and 6C). We have not looked at the contribution of increased expression of glucose transporters, hexokinase or the direct effect of inflammatory mediators to the increased NIR 2-DG retention noted in the GTL group. However the *in vitro* data in isolated acinar cells showing increased NIR 2-DG retention within an hour of GTL exposure, in the absence of exogenous inflammatory mediators (Fig 5A and 5B) makes the contribution of these to the *in vivo* signal unlikely.

These studies make a case that measuring the increase in metabolic demand of the pancreas helps predict the subsequent severity of local injury. This is important since most clinical cases of AP are mild, and currently there are no reliable early predictors to distinguish these mild ones from the ones that subsequently develop severe necrosis. In the clinical setting necrosis typically develops after the first several days of AP, and an early predictor of necrosis may allow early changes in management and help focus therapeutic approaches to patients with predicted severe AP.

In summary, the current studies show increased and persistent accumulation of the near-infrared 2-DG probe, IRDye^®^ 800CW 2-DG, over the pancreas during the initial hours of AP to be a more reliable predictor of severity than conventional serum markers of AP. This binding of the 2-DG probe precedes the drop in ATP levels and necrosis that eventually results in severe AP. Based on the utility demonstrated in this rapidly evolving inflammatory disease model in rats, this study opens the scope of imaging 2-DG analogs as early predictors of inflammatory disease severity in humans.

## References

[1] Saboury B, Parsons MA, Moghbel M, Rubello D, Brothers A, et al (2015) Quantification of aging effects upon global knee inflammation by 18F-FDG-PET. Nuclear medicine communications. 10.1097/MNM.0000000000000430 26555103

[2] Okuyucu K, Alagoz E, Demirbas S, Ince S, Karakas A, et al (2015) Evaluation of predictor variables of diagnostic [18F] FDG-PET/CT in fever of unknown origin. The quarterly journal of nuclear medicine and molecular imaging: official publication of the Italian Association of Nuclear Medicine. 10.23736/S1824-4785.17.02833-3 26554525

[3] Albano D, Bosio G, Bertagna F (2015) Mesenteric Panniculitis Demonstrated on 18F-FDG PET/CT. Clinical nuclear medicine. 10.1097/RLU.0000000000001056 26462046

[4] Kang WJ (2015) F-18 Fluoride Positron Emission Tomography-Computed Tomography for Detecting Atherosclerotic Plaques. Korean journal of radiology 16: 1257–1261. 10.3348/kjr.2015.16.6.1257 26576114 PMC4644746

[5] Folco EJ, Sheikine Y, Rocha VZ, Christen T, Shvartz E, et al (2011) Hypoxia but not inflammation augments glucose uptake in human macrophages: Implications for imaging atherosclerosis with 18fluorine-labeled 2-deoxy-D-glucose positron emission tomography. Journal of the American College of Cardiology 58: 603–614. 10.1016/j.jacc.2011.03.044 21798423

[6] Kato K, Nihashi T, Ikeda M, Abe S, Iwano S, et al (2013) Limited efficacy of (18)F-FDG PET/CT for differentiation between metastasis-free pancreatic cancer and mass-forming pancreatitis. Clinical nuclear medicine 38: 417–421. 10.1097/RLU.0b013e3182817d9d 23486318

[7] Dong A, Dong H, Zhang L, Zuo C (2013) Hypermetabolic lesions of the pancreas on FDG PET/CT. Clinical nuclear medicine 38: e354–366. 10.1097/RLU.0b013e3182708503 23486316

[8] Pery C, Meurette G, Ansquer C, Frampas E, Regenet N (2010) Role and limitations of 18F-FDG positron emission tomography (PET) in the management of patients with pancreatic lesions. Gastroenterologie clinique et biologique 34: 465–474. 10.1016/j.gcb.2009.04.014 20688444

[9] Ozaki Y, Oguchi K, Hamano H, Arakura N, Muraki T, et al (2008) Differentiation of autoimmune pancreatitis from suspected pancreatic cancer by fluorine-18 fluorodeoxyglucose positron emission tomography. Journal of gastroenterology 43: 144–151. 10.1007/s00535-007-2132-y 18306988

[10] Yokoyama Y, Nagino M, Hiromatsu T, Yuasa N, Oda K, et al (2005) Intense PET signal in the degenerative necrosis superimposed on chronic pancreatitis. Pancreas 31: 192–194. 16025008 10.1097/01.mpa.0000168226.36085.58

[11] Imdahl A, Nitzsche E, Krautmann F, Hogerle S, Boos S, et al (1999) Evaluation of positron emission tomography with 2-[18F]fluoro-2-deoxy-D-glucose for the differentiation of chronic pancreatitis and pancreatic cancer. The British journal of surgery 86: 194–199. 10100786 10.1046/j.1365-2168.1999.01016.x

[12] Kovar JL, Volcheck W, Sevick-Muraca E, Simpson MA, Olive DM (2009) Characterization and performance of a near-infrared 2-deoxyglucose optical imaging agent for mouse cancer models. Analytical biochemistry 384: 254–262. 10.1016/j.ab.2008.09.050 18938129 PMC2720560

[13] Kovar JL, Simpson MA, Schutz-Geschwender A, Olive DM (2007) A systematic approach to the development of fluorescent contrast agents for optical imaging of mouse cancer models. Analytical biochemistry 367: 1–12. 17521598 10.1016/j.ab.2007.04.011

[14] Durgampudi C, Noel P, Patel K, Cline R, Trivedi RN, et al (2014) Acute Lipotoxicity Regulates Severity of Biliary Acute Pancreatitis without Affecting Its Initiation. The American journal of pathology 184: 1773–1784. 10.1016/j.ajpath.2014.02.015 24854864 PMC4044711

[15] Le T, Eisses JF, Lemon KL, Ozolek JA, Pociask DA, et al (2015) Intraductal infusion of taurocholate followed by distal common bile duct ligation leads to a severe necrotic model of pancreatitis in mice. Pancreas 44: 493–499. 10.1097/MPA.0000000000000285 25469547 PMC4357535

[16] Fitz RH (1889) Acute pancreatitis: a consideration of pancreatic hemorrhage, hemorrhagic, suppurative, and gangrenous pancreatitis, and of disseminated fat-necrosis. Boston: Cupples and Hurd 91 p. p.

[17] Hotchkiss LW (1912) VIII. Acute Pancreatitis with Very Extensive Fat Necrosis. Annals of surgery 56: 111–117. 17862860 10.1097/00000658-191207000-00009 PMC1407312

[18] Kloppel G, von Gerkan R, Dreyer T (1984) Pathomorphology of acute pancreatitis Analysis of 367 autopsy cases and 3 surgical specimens; Gyr KE S M, Sarles H, editor. Amsterdam, New York, Oxford.

[19] Renner IG, Savage WT III, Pantoja JL, Renner VJ (1985) Death due to acute pancreatitis. A retrospective analysis of 405 autopsy cases. Digestive diseases and sciences 30: 1005–1018. 3896700 10.1007/BF01308298

[20] Aho HJ, Sternby B, Nevalainen TJ (1986) Fat necrosis in human acute pancreatitis. An immunohistological study. Acta pathologica, microbiologica, et immunologica Scandinavica Section A, Pathology 94: 101–105. 3521189 10.1111/j.1699-0463.1986.tb02970.x

[21] Nordback I, Lauslahti K (1986) Clinical pathology of acute necrotising pancreatitis. Journal of clinical pathology 39: 68–74. 3950033 10.1136/jcp.39.1.68 PMC499615

[22] Navina S, Acharya C, DeLany JP, Orlichenko LS, Baty CJ, et al (2011) Lipotoxicity causes multisystem organ failure and exacerbates acute pancreatitis in obesity. Science translational medicine 3: 107ra110 10.1126/scitranslmed.3002573 22049070 PMC3321362

[23] Schmitz-Moormann P (1981) Comparative radiological and morphological study of the human pancreas. IV. Acute necrotizing pancreatitis in man. Pathol Res Pract 171: 325–335. 7279784 10.1016/S0344-0338(81)80105-7

[24] Schmitz-Moormann P, Pittner PM, Heinze W (1981) Lipomatosis of the pancreas. A morphometrical investigation. Pathol Res Pract 173: 45–53. 7335549 10.1016/S0344-0338(81)80006-4

[25] Saisho Y, Butler AE, Meier JJ, Monchamp T, Allen-Auerbach M, et al (2007) Pancreas volumes in humans from birth to age one hundred taking into account sex, obesity, and presence of type-2 diabetes. Clin Anat 20: 933–942. 17879305 10.1002/ca.20543 PMC2680737

[26] Pinnick KE, Collins SC, Londos C, Gauguier D, Clark A, et al (2008) Pancreatic ectopic fat is characterized by adipocyte infiltration and altered lipid composition. Obesity (Silver Spring) 16: 522–530. 18239594 10.1038/oby.2007.110

[27] Singh VP, McNiven MA (2008) Src-mediated cortactin phosphorylation regulates actin localization and injurious blebbing in acinar cells. Mol Biol Cell 19: 2339–2347. 10.1091/mbc.E07-11-1130 18353971 PMC2366849

[28] Mishra V, Patel K, Trivedi RN, Noel P, Durgampudi C, et al (2014) Hypothermia slows sequential and parallel steps initiated during caerulein pancreatitis. Pancreatology: official journal of the International Association of Pancreatology 14: 459–464. 10.1016/j.pan.2014.06.006 25459565

[29] Mishra V, Cline R, Noel P, Karlsson J, Baty CJ, et al (2013) Src Dependent Pancreatic Acinar Injury Can Be Initiated Independent of an Increase in Cytosolic Calcium. PloS one 8: e66471 23824669 10.1371/journal.pone.0066471 PMC3688910

[30] Acharya C, Navina S, Singh VP (2014) Role of pancreatic fat in the outcomes of pancreatitis. Pancreatology: official journal of the International Association of Pancreatology 14: 403–408. 10.1016/j.pan.2014.06.004 PMC4185152 25278311

[31] Noel P, Patel K, Durgampudi C, Trivedi RN, de Oliveira C, et al (2014) Peripancreatic fat necrosis worsens acute pancreatitis independent of pancreatic necrosis via unsaturated fatty acids increased in human pancreatic necrosis collections. Gut. 10.1136/gutjnl-2014-308043 PMC4869971 25500204

[32] Patel K, Trivedi RN, Durgampudi C, Noel P, Cline RA, et al (2015) Lipolysis of visceral adipocyte triglyceride by pancreatic lipases converts mild acute pancreatitis to severe pancreatitis independent of necrosis and inflammation. The American journal of pathology 185: 808–819. 10.1016/j.ajpath.2014.11.019 25579844 PMC4348470

[33] Acharya C, Cline RA, Jaligama D, Noel P, Delany JP, et al (2013) Fibrosis Reduces Severity of Acute-on-Chronic Pancreatitis in Humans. Gastroenterology 145: 466–475. 10.1053/j.gastro.2013.05.012 23684709 PMC3964816

[34] (2013) IAP/APA evidence-based guidelines for the management of acute pancreatitis. Pancreatology: official journal of the International Association of Pancreatology 13: e1–15. 10.1016/j.pan.2013.07.063 24054878

[35] Zhou H, Luby-Phelps K, Mickey BE, Habib AA, Mason RP, et al (2009) Dynamic near-infrared optical imaging of 2-deoxyglucose uptake by intracranial glioma of athymic mice. PloS one 4: e8051 10.1371/journal.pone.0008051 19956682 PMC2778127

[36] Wu JC, Nguyen PK (2011) Imaging atherosclerosis with F18-fluorodeoxyglucose positron emission tomography: What are we actually seeing? Journal of the American College of Cardiology 58: 615–617. 10.1016/j.jacc.2011.04.021 21798424

[37] Kovar JL, Xu X, Draney D, Cupp A, Simpson MA, et al (2011) Near-infrared-labeled tetracycline derivative is an effective marker of bone deposition in mice. Analytical biochemistry 416: 167–173. 10.1016/j.ab.2011.05.011 21645491

[38] Panek J, Sztefko K, Drozdz W (2001) Composition of free fatty acid and triglyceride fractions in human necrotic pancreatic tissue. Med Sci Monit 7: 894–898. 11535930

[39] Mukherjee R, Criddle DN, Gukovskaya A, Pandol S, Petersen OH, et al (2008) Mitochondrial injury in pancreatitis. Cell calcium 44: 14–23. 10.1016/j.ceca.2007.11.013 18207570 PMC6663091

[40] Petersen OH, Tepikin AV, Gerasimenko JV, Gerasimenko OV, Sutton R, et al (2009) Fatty acids, alcohol and fatty acid ethyl esters: toxic Ca2+ signal generation and pancreatitis. Cell Calcium 45: 634–642. 10.1016/j.ceca.2009.02.005 19327825

[41] Aho HJ, Koskensalo SM, Nevalainen TJ (1980) Experimental pancreatitis in the rat. Sodium taurocholate-induced acute haemorrhagic pancreatitis. Scandinavian journal of gastroenterology 15: 411–416. 7433903 10.3109/00365528009181493

[42] Aho HJ, Nevalainen TJ, Lindberg RL, Aho AJ (1980) Experimental pancreatitis in the rat. The role of phospholipase A in sodium taurocholate-induced acute haemorrhagic pancreatitis. Scandinavian journal of gastroenterology 15: 1027–1031. 6165066 10.3109/00365528009181808

[43] Nioka S, Chance B (2005) NIR spectroscopic detection of breast cancer. Technology in cancer research & treatment 4: 497–512. 16173821 10.1177/153303460500400504

